# Mutations at amino-acid 482 in the *ABCG*2 gene affect substrate and antagonist specificity

**DOI:** 10.1038/sj.bjc.6601370

**Published:** 2003-11-11

**Authors:** R W Robey, Y Honjo, K Morisaki, T A Nadjem, S Runge, M Risbood, M S Poruchynsky, S E Bates

**Affiliations:** 1 National Institutes of Health, Center for Cancer Research, Cancer Therapeutics Branch, Bethesda, MD 20892, USA

**Keywords:** drug-resistance, BCRP/MXR/ABCP/ABCG2, mitoxantrone, flow cytometry, mutation

## Abstract

Recent studies have shown that mutations at amino-acid 482 in the *ABCG2* gene affect the substrate specificity of the protein. To delineate the effects of these mutations clearly, human embryonic kidney cells (HEK-293) were stably transfected with wild-type 482R or mutant 482G and 482T ABCG2. By flow cytometry, mitoxantrone, BODIPY-prazosin, and Hoechst 33342 were found to be substrates of all ABCG2 proteins, while rhodamine 123, daunorubicin, and LysoTracker Green were transported only by mutant ABCG2. In cytotoxicity assays, all ABCG2 proteins conferred high levels of resistance to mitoxantrone, SN-38, and topotecan, while mutant ABCG2 also exhibited a gain of function for mitoxantrone as they conferred a four-fold greater resistance compared to wild type. Cells transfected with mutant ABCG2 were 13- to 71- fold resistant to the P-glycoprotein substrates doxorubicin, daunorubicin, epirubicin, bisantrene, and rhodamine 123 compared to cells transfected with wild-type ABCG2, which were only three- to four-fold resistant to these compounds. ABCG2 did not confer appreciable resistance to etoposide, taxol or the histone deacetylase inhibitor depsipeptide. None of the transfected cell lines demonstrated resistance to flavopiridol despite our previous observation that ABCG2-overexpressing cell lines are cross-resistant to the drug. Recently reported inhibitors of ABCG2 were evaluated and 50 *μ*M novobiocin was found to reverse wild-type ABCG2 completely, but only reverse mutant ABCG2 partially. The studies presented here serve to underscore the importance of amino-acid 482 in defining the substrate specificity of the ABCG2 protein and raise the possibility that amino-acid 482 mutations in human cancers could affect the clinical application of antagonists for ABCG2.

ABCG2 is an ATP-binding cassette half-transporter (Allikmets *et al*, 1998; Doyle *et al*, 1998; Miyake *et al*, 1999) that has been shown to confer resistance to a variety of chemotherapeutic agents including mitoxantrone (Brangi *et al*, 1999; Litman *et al*, 2000); the camptothecins topotecan (Maliepaard *et al*, 1999; Yang *et al*, 2000) and SN-38 (Kawabata *et al*, 2001); doxorubicin (Chen *et al*, 1990); and flavopiridol (Robey *et al*, 2001b). Cells overexpressing wild-type ABCG2 with an arginine at amino-acid 482 have been shown by flow cytometric analysis to transport mitoxantrone, while those overexpressing ABCG2 with a threonine or glycine at position 482 (R482G, R482T) transported mitoxantrone and also exhibited a gain in function with the transport of rhodamine 123 and daunorubicin (Honjo *et al*, 2001). In Cytotoxicity assays, cells overexpressing any of the ABCG2 proteins are resistant to mitoxantrone, topotecan, and SN-38, while cells overexpressing mutant ABCG2 are additionally resistant to the anthracyclines. Although these phenotypic differences have been well described in selected cell lines, careful studies in transfected cells were needed to exclude bias due to other mechanisms of resistance in selected cell lines.

A similar situation was recently described for the mouse homologue, Abcg2. Allen *et al* (1999) reported overexpression of Abcg2 in mouse fibroblast cell lines lacking functional Mdr1a, Mdr1b, and Mrp1, which were selected in mitoxantrone, doxorubicin, or topotecan. Greater doxorubicin resistance was observed in the cells selected in doxorubicin compared to those selected in mitoxantrone or topotecan. Upon sequencing Abcg2 in these cell lines and two other fibroblast cell lines selected in doxorubicin, it was noted that the arginine at position 482 in wild-type Abcg2 was mutated to a methionine or serine. Expression of the mutant Abcg2 proteins conferred greater resistance to the anthracyclines as well as the ability to transport rhodamine 123 (Allen *et al*, 2002). Allen suggested that the 482 residue represented a hot spot for mutation in ABCG2. Subsequent to these studies, an arginine to methionine mutation was also described in a selected human cell line (Wang *et al*, 2003).

To characterize precisely the cross-resistance profile conferred by the wild-type and mutant ABCG2 proteins, we transfected human embryonic kidney (HEK-293) cells with 482R, 482T, or 482G ABCG2. In transfected cell lines expressing comparable levels of ABCG2, we tested the ability of the transfectants to transport fluorescent compounds reported to be substrates of the half-transporter, and performed killing curves with chemotherapeutic agents reported to be effluxed by ABCG2 to determine the cross-resistance profile conferred by each of the ABCG2 proteins.

The interactions of the recently reported ABCG2 antagonists novobiocin (Doyle *et al*, 2002; Shiozawa *et al*, 2002), *β*-estradiol, and estrone (Imai *et al*, 2002) with wild-type and mutant ABCG2 were also examined. The results presented here demonstrate amino-acid 482 to be significant in determining the effectiveness of ABCG2 antagonists as well as the ability to transport selected antineoplastic drugs.

## MATERIALS AND METHODS

### Chemicals

The fluorescent compounds LysoTracker Green DND-26 and BODIPY-prazosin were purchased from Molecular Probes (Eugene, OR, USA). Flavopiridol, topotecan, depsipeptide, and epirubicin were obtained from the National Cancer Institute Drug Screen. Mitoxantrone, daunorubicin, doxorubicin, Hoechst 33342, rhodamine 123, novobiocin, estrone, and *β*-estradiol were purchased from Sigma Chemical Co. (St Louis, MO, USA). Bisantrene was a gift from Dr Lee Greenberger (Wyeth-Ayerst). Fumitremorgin C (FTC) was synthesised by Thomas McCloud, Developmental Therapeutics Program, Natural Products Extraction Laboratory, National Institutes of Health (Bethesda, MD, USA).

### Establishment of stable transfectants

HEK-293 cells were transfected with either empty pcDNA3 vector (Invitrogen, Carlsbad, CA, USA) or pcDNA3 vector containing full-length ABCG2 coding either an arginine, threonine or glycine for amino-acid 482. Expression of ABCG2 in the transfectants was enforced by selection in G418 (Invitrogen, Carlsbad, CA, USA). Stable transfectants were maintained in Eagle's minimum essential medium (ATCC, Manassas, VA, USA) supplemented with 10% FCS, penicillin, and streptomycin with G418 at a concentration of 2 mg ml^−1^. Clones were preliminarily screened for ABCG2 expression by examining the ability of the cells to efflux BODIPY-prazosin in a flow cytometry-based assay. The ABCG2 sequence was subsequently verified in the clones examined here.

### RNA isolation and Northern blot analysis

RNA was extracted from cells using RNA STAT-60 (Tel-Test Inc., Friendswood, TX, USA) according to the manufacturer's instructions. Northern blot analysis was performed using a riboprobe generated from the first 662 bp of ABCG2 subcloned into a pCRII-TOPO vector (Invitrogen, Carlsbad, CA, USA).

### Western blot analysis

Microsomal membrane fractions (30 *μ*g) were subjected to electrophoresis and transferred to nitrocellulose membranes as previously described (Robey *et al*, 2001b). Blots were probed with the monoclonal anti-BCRP antibody BXP-21 (Maliepaard *et al*, 2001) (Kamiya Biomedical, Seattle WA, USA) as previously described (Litman *et al*, 2000).

### Flow cytometry

Flow cytometry assays were performed as previously described (Robey *et al*, 2001a). Briefly, cells were trypsinised, resuspended in complete media (phenol red-free IMEM with 10% fetal calf serum) containing 20 *μ*M mitoxantrone, 5 *μ*g ml^−1^ daunorubicin, 250 nM BODIPY-prazosin, 0.5 *μ*g ml^−1^ rhodamine 123, 250 nM LysoTracker Green DND-26, or 10 *μ*M Hoechst 33342 with or without 10 *μ*M of the ABCG2 blocker, FTC, and incubated for 30 min at 37°C in 5% CO_2_. FTC has been reported to be a specific blocker of ABCG2 (Rabindran *et al*, 1998, Rabindran *et al*, 2000), and we assumed 10 *μ*M FTC afforded complete prevention of ABCG2-mediated efflux based on previous results. In studies with reported ACBG2 antagonists, cells were incubated with 250 nM BODIPY-prazosin alone or with various concentrations of the inhibitors. Cells were then washed once in cold complete medium and then incubated for 1 h at 37°C in substrate-free media continuing with or without 10 *μ*M FTC or the described concentrations of the other ABCG2 inhibitors to generate the efflux and FTC/efflux histograms (or inhibitor/efflux histograms), respectively. Subsequently, cells were washed twice with cold DPBS and placed on ice in the dark until analysed. FTC/efflux–efflux values, calculated as the difference in mean channel numbers between the FTC/efflux and efflux peaks, were generated for each cell line with each fluorescent substrate. Cells were analysed either on a FACSort flow cytometer equipped with both a 488 nm argon laser and a 635 nm red diode laser or a FACSVantage flow cytometer equipped with a 360 nM UV laser to detect Hoechst 33342 fluorescence. For all samples, at least 10 000 events were collected. Debris was eliminated by gating on forward *vs* side scatter and dead cells were excluded based on propidium iodide staining.

For studies with the anti-ABCG2 antibody, 5D3, cells were trypsinised and resuspended in DPBS with 2% BSA to which was added phycoerythrin-conjugated 5D3 (eBioscience, San Diego, CA, USA) or phycoerythrin-conjugated mouse IgG. The cells were incubated with antibody for 30 min at room temperature, washed twice with DPBS and kept in the dark until analysed.

### Cytotoxicity assays

The cytotoxocity assays performed were based on the previously described sulphorhodamine B assay (Skehan *et al*, 1990). Cells were plated in flat-bottom 96-well plates at a density of 2000 cells per well and allowed to attach for 24 h at 37°C in 5% CO_2_. Chemotherapeutic agents at various concentrations were added to the cells and the plates were allowed to incubate for 96 h at 37°C in 5% CO_2_. Subsequently, cells were fixed in 12.5% trichloroacetic acid and then stained with sulphorhodamine B solution (0.4% sulphorhodamine B w v^−1^ in 1% acetic acid). Optical densities were read on a Bio-Rad plate reader at an absorbance of 540 nm. Each concentration was tested in triplicate and controls were performed in replicates of eight.

## RESULTS

### Expression of ABCG2 in HEK-293 cells

**Figure 1:**
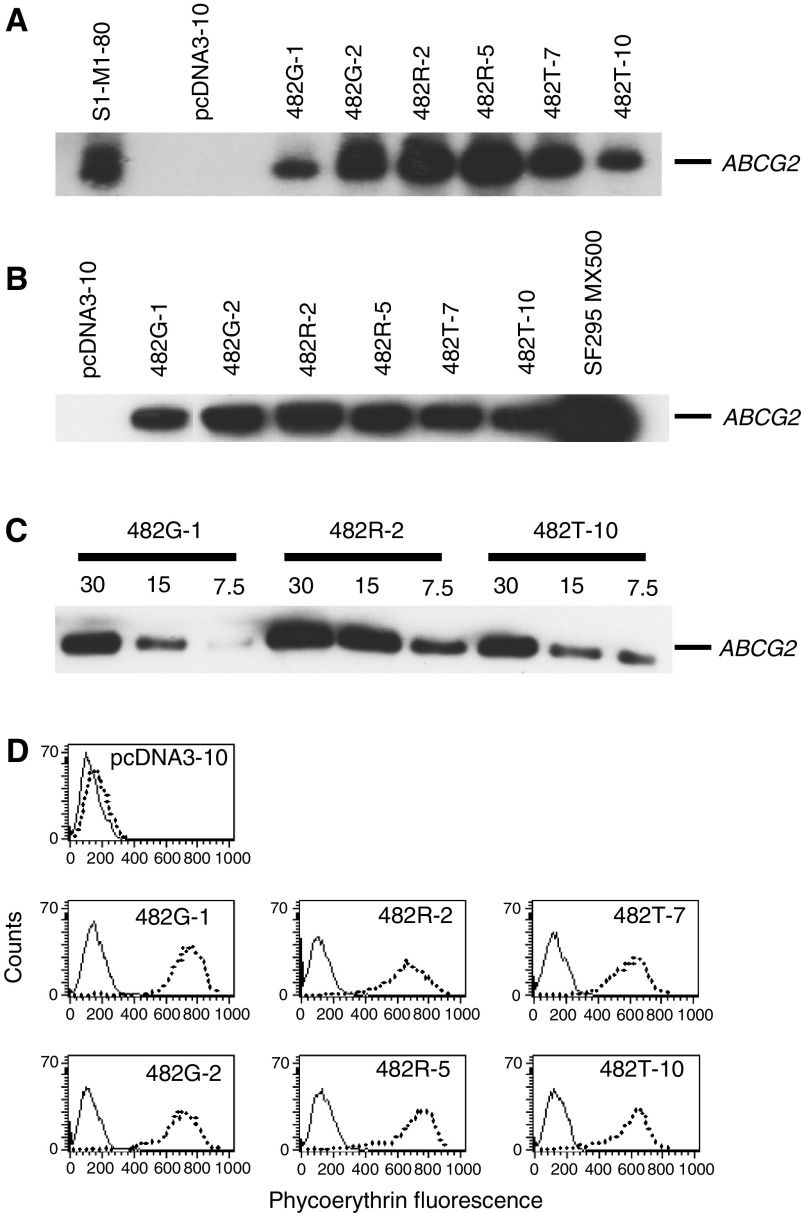
Expression of ABCG2 in HEK-293 cells transfected with wild-type and mutant ABCG2. (**A**) Northern blot analysis was performed on 20 *μ*g RNA extracted from six cell lines transfected with ABCG2 (482G-1, 482G-2, 482R-2, 482R-5, 482T-7, 482T-10) and one cell line transfected with empty vector (pcDNA3-10) and probed with a riboprobe generated from the first 662 bp of ABCG2. (**B**) Immunoblot analysis for ABCG2 using the anti-ABCG2 antibody BXP-21 was performed on membrane protein (30 *μ*g) from the seven transfected cell lines. (**C**) Immunoblot analysis for ABCG2 with serial dilutions of membranes isolated from same transfected clones (482G-1, 482R-2, 482T-10) subsequently used in cytotoxicity assays. (**D**) The seven transfected cell lines were incubated for 30 min with either phycoerythrin-labelled negative control antibody (dotted line) or phycoerythrin-labelled anti-ABCG2 antibody (dashed line) and then analysed on a flow cytometer. Representative histograms are shown.

HEK-293 cells were transfected with the ABCG2 gene and screened based on BODIPY-prazosin transport (data not shown). From the positive clones obtained, two clones transfected with each ABCG2 gene (482G-1, 482G-2, 482R-2, 482R-5, 482T-7, 482T-10) and one clone transfected with empty vector (pcDNA3-10) were selected and RNA was extracted. Northern blotting was performed using 20 *μ*g total RNA. As shown in Figure 1A

, all clones transfected with ABCG2 have enforced expression of the ABCG2 gene at the RNA level; in the cell line transfected with empty vector, no ABCG2 RNA was detected. Western blotting was performed on 30 *μ*g membrane protein isolated from the seven cell lines and comparable protein expression was observed (Figure 1B). Serial dilutions of protein from the transfectants used in cytotoxicity assays revealed about a two-fold higher level in wild-type-expressing cells (482R-2) than the mutant-expressing cells (482G-1, 482T-10) as seen in Figure 1C. The seven cell lines shown in Figure 1A were then incubated with an anti-ABCG2 monoclonal antibody, clone 5D3 (Zhou *et al*, 2001), to measure the expression of ABCG2 on the cell surface by flow cytometry. All stably transfected cell lines demonstrated comparable expression of ABCG2 on the cell surface, with the empty vector transfected cells expressing no ABCG2 (Figure 1D); the difference in channel numbers between cells incubated with negative control antibody and the anti-ABCG2 antibody 5D3 are given in Table 1

**Table 1:** Antibody labelling and FTC-inhibitable efflux of fluorescent compounds in ABCG2 transfected cell lines

**Cell line**	**5D3^a^**	**BODIPY-prazosin^b^**	**Mitoxantrone^b^**	**LysoTracker**^b^	**Daunorubicin^b^**	**Hoechst 33342^b^**	**Rhodamine 123^b^**
482G-1	531.0±42.6	314.2±21.4	183.3±16.6	91.3±2.1	117.9±1.2	208.0±12.7	241.9±18.9
482G-2	504.2±41.3	294.7±26.1	181.9±24.3	77.7±10.0	137.36±10.2	234.8±8.2	214.9±27.1
482R-2	474.7±51.6	186.9±41.2	120.6±16.5	18.4±10.4	4.4±0.4	164.8±4.3	0
482R-5	557.2±16.4	233.3±13.6	162±9.8	21.5±14.7	12.4±0.6	212.1±16.5	0
482T-7	443.5±30.1	259.6±18.8	137.3±25.8	75.7±8.6	128.98±14.6	216.7±3.3	238.2±13.2
482T-10	471.4±18.6	223.3±34.9	143.8±25.4	85.39±16.2	166.3±6.9	199.6±5.1	232.4±28.2

a The values calculated are the difference in mean channel numbers between the 5D3 antibody histogram and the negative control antibody histogram.

b Difference in mean channel number between the FTC/efflux histogram and the efflux histogram. Negative values were assigned the value of zero. Values obtained were from at least two independent experiments.

. Despite higher ABCG2 expression at the RNA level in the two 482R clones, similar levels of ABCG2 protein were found in all of the transfectants. The sequence of the transfected ABCG2 was confirmed in the six positive clones.

### Transport of fluorescent substrates by ABCG2-transfected HEK-293 cells

**Figure 2:**
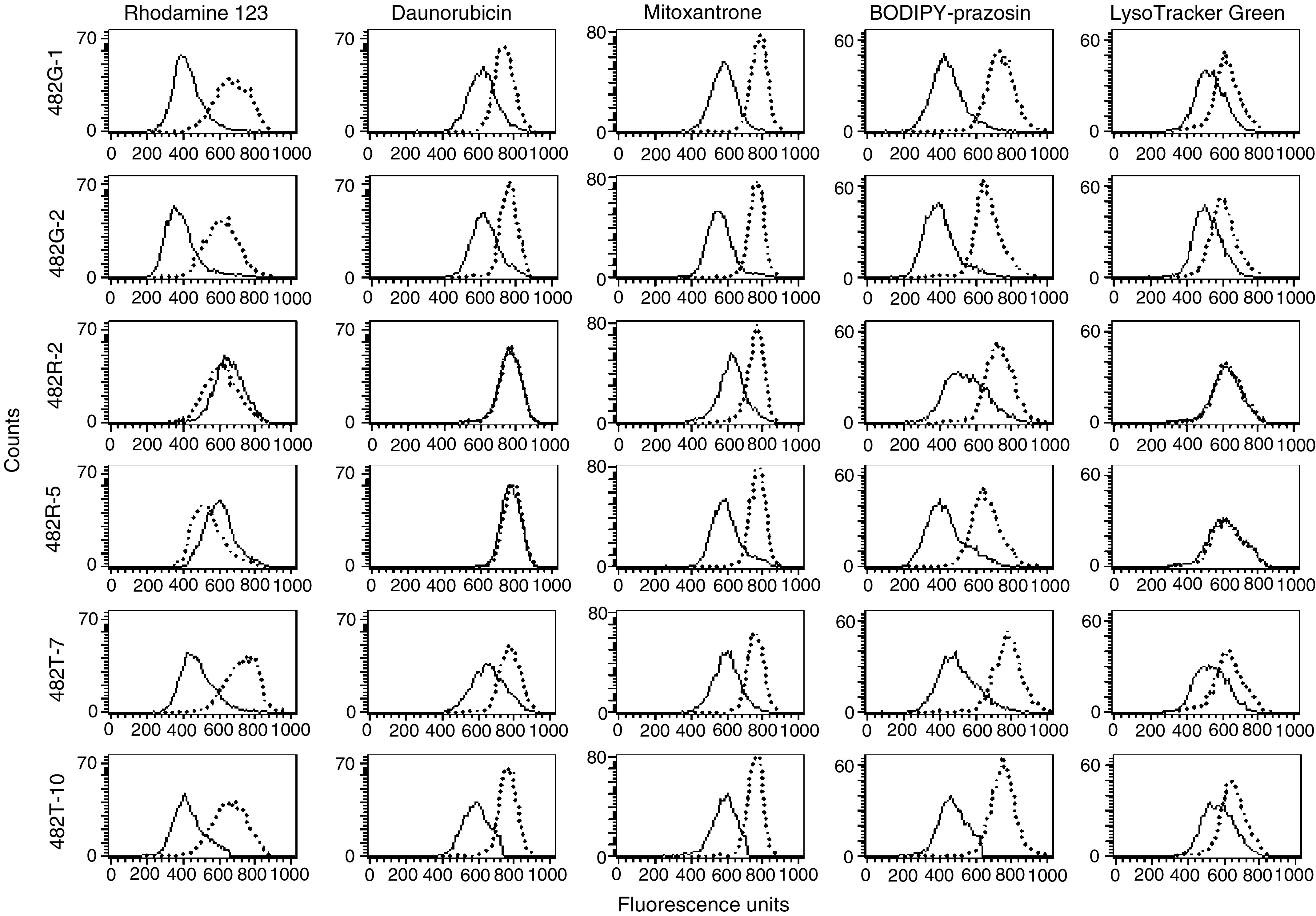
Transport of fluorescent substrates by wild-type and mutant ABCG2. The six transfected cell lines were incubated in rhodamine 123 (0.5 *μ*g ml^−1^), daunorubicin (5 *μ*g ml^−1^), mitoxantrone (20 *μ*M), BODIPY-prazosin (250 nM), or LysoTracker Green (250 nM) with or without 10 *μ*M FTC for 30 min at 37°C. Cells were then washed, allowed to efflux for 1 h at 37°C in substrate-free media continuing with (dotted line) or without (solid line) FTC, and analysed on a flow cytometer. Representative results are shown. Mean channel differences from 2 or more experiments are present in Table 1.

The amino acid at position 482 has been found to be predictive for the ability of ABCG2 to transport fluorescent substrates (Honjo *et al*, 2001). Representative histograms for rhodamine 123, daunorubicin, mitoxantrone, BODIPY-prazosin, and LysoTracker Green are shown in Figure 2

. Here the dotted lines represented the accumulation of the substrate in the presence of the ABCG2 inhibitor fumitremorgin C, while the solid line represents the accumulation without inhibitor. Reduced accumulation that is increased by FTC is present to varying degrees in the 482G and 482T mutants for all of the substrates. In contrast, those vectors carrying the wild-type (482R) ABCG2 showed evidence of transport only of mitoxantrone and BODIPY-prazosin. FTC/efflux–efflux values for BODIPY-prazosin, rhodamine 123, daunorubicin, LysoTracker Green DND-26, and mitoxantrone were calculated for each cell line. We have previously shown that FTC-inhibitable mitoxantrone or BODIPY-prazosin efflux (FTC/efflux–efflux value) is proportional to the expression of ABCG2 in cell lines (Robey *et al*, 2001a). The results are summarised in Table 1; negative FTC/efflux–efflux values were given the value of zero. FTC/efflux–efflux values for mitoxantrone and BODIPY-prazosin were comparable among the transfected cell lines. However, efflux of rhodamine 123, LysoTracker Green and daunorubicin is only observed in HEK-293 cells transfected with mutant 482G or 482T ABCG2. As the dye Hoechst 33342 has recently been shown to be a substrate of ABCG2 (Zhou *et al*, 2001; Kim *et al*, 2002; Scharenberg *et al*, 2002), the ability of the three ABCG2 proteins to transport the compound was evaluated and confirmed for all three proteins. None of the fluorescent compounds was transported by the cell line transfected with empty vector (data not shown).

### Sensitivity of HEK-293 cells transfected with wild-type or mutant ABCG2 to antineoplastic agents

The 4-day cytotoxicity assays were performed on the empty vector transfected cells as well as the 482G-1, 482R-2, and 482T-10 transfected clones. The results are summarised in Table 2;

**Table 2:** Cross-resistance profile of HEK-293 cells transfected with wild-type and mutant ABCG2

	**pcDNA3**	**482G-1**		**482R-2**		**482T-10**	
**Drug**	**IC50 (nM)**	**IC50 (nM)**	**RR^a^**	**IC50 (nM)**	**RR**	**IC50 (nM)**	**RR**
Mitoxantrone	3.8±1.8	543±53	140	107±64	28	457±287	118
Topotecan	8±2	175±50	22	275±50	34	167±65	21
SN-38	1.2±1.1	65±35	52	120±113	96	60±42	48
Doxorubicin	11.2±6.3	575±171	51	35±13	3	800±245	71
Daunorubicin	20±0.1	200±1	10	40±1	2	333±58	17
Bisantrene	127±64	1667±577	13	633±115	5	1667±577	13
Epirubicin	25±7.1	600±141	24	95±7.1	4	700±424	28
Paclitaxel	9±1	20±1	2	30±14	3	30±1	3
Depsipeptide	4±1	6±1	1.5	8±1	2	7.5±1	2
Flavopiridol	123±68	200±10	2	160±69	1	200±10	2
Etoposide	75±7	650±71	9	250±71	3	450±212	6
Rhodamine 123	7000±2000	267000±115000	38	53000±12000	8	267000±58000	38

a Relative resistance values were obtained by dividing the IC_50_ value of the ABCG2-transfected cell lines by the IC_50_ value of the empty vector transfected cell line. Values obtained were from two to five separate experiments.

representative curves for some of the compounds tested are shown in Figure 3

**Figure 3:**
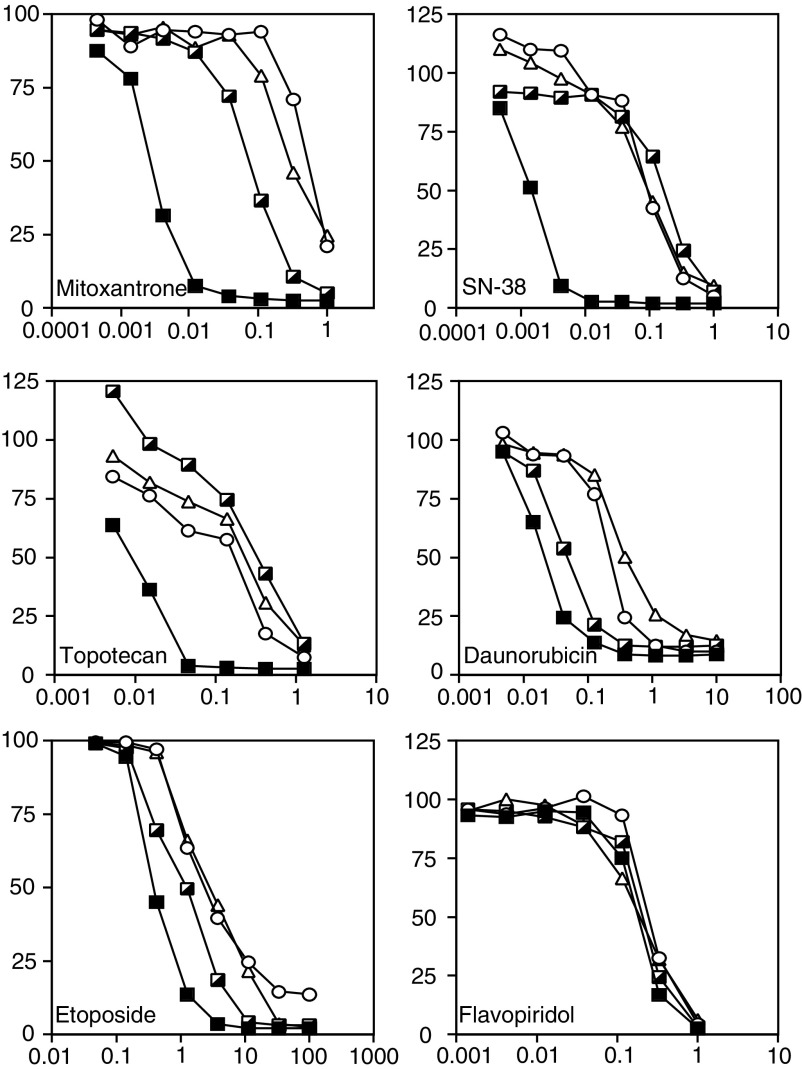
Cross-resistance profile conferred by wild-type and mutant ABCG2. The 4-day cytotoxicity assays were performed with mitoxantrone, SN-38, topotecan, daunorubicin, etoposide, and flavopiridol on HEK-293 cells transfected with empty vector (filled squares), or transfected with 482R (hatched squares), 482G (open circles), or 482 T (open triangles) ABCG2. Curves representative of two to five separate experiments are shown.

. In agreement with previous reports, HEK-293 cells transfected with any of the ABCG2 genes exhibited high levels of resistance to mitoxantrone compared to empty vector-transfected cells; however, IC_50_ values for 482G-1 and 482T-10, 543 and 457 nM respectively, were about four-fold higher than for the 482R-2 cells, 107 nM. When cytotoxicity experiments were performed with mitoxantrone on another set of clones, 482G-2, 482R-5, and 482T-7, the HEK-293 cells expressing either of the mutant ABCG2 proteins (482G-2, 482T-7) had IC_50_ values that were again about four-fold higher than for cells expressing wild-type 482R-5 ABCG2 (data not shown). Both sets of experiments suggest that mitoxantrone is a better substrate for mutant ABCG2 than wild type. HEK-293 cells expressing any of the ABCG2 proteins demonstrated 21- to 34-fold resistance to topotecan and 48- to 96-fold resistance to SN-38. For either drug, the IC_50_ of the 482R wild-type clone was higher than that observed in the clones expressing mutant ABCG2. Cells transfected with wild-type ABCG2 showed little resistance to the anthracyclines doxorubicin and daunorubicin, as well as bisantrene, epirubicin, and rhodamine 123, compared to mutant ABCG2. ABCG2 conferred low levels of resistance to etoposide, but did not confer obvious resistance to depsipeptide or paclitaxel. Surprisingly, none of the ABCG2 proteins conferred appreciable resistance to flavopiridol. This is in contrast to a previous finding showing resistance to flavopiridol in selected cell lines overexpressing ABCG2 and overexpression of ABCG2 in cell lines selected in flavopiridol (Robey *et al*, 2001b).

### Effect of amino-acid 482 mutations on antagonist efficacy

As mutations in Pgp have been shown to affect the ability of Pgp antagonists to prevent Pgp-mediated efflux (Chen *et al*, 1997; Ma *et al*, 1997; Watanabe *et al*, 2000), we examined the impact of the mutations at amino-acid 482 on ABCG2 antagonists. ABCG2-transfected cells were incubated for 30 min in 250 nM BODIPY-prazosin alone, or with 50 *μ*M novobiocin, 100 *μ*M novobiocin, 100 *μ*M *β*-estradiol, 100 *μ*M estrone, or 10 *μ*M FTC. Cells were then washed and incubated for 1 h in substrate-free media continuing without or with the desired blocker to generate the efflux or inhibitor/efflux histograms, respectively. The FTC/efflux histogram obtained from cells incubated in 10 *μ*M FTC was considered maximal accumulation. The values in Table 3

**Table 3:** Inhibition of BODIPY-prazosin efflux in ABCG2 transfectants assayed by flowcytometry

	**100 *μ*M *β*-estradiol^a^**	**100 *μ*M estrone^a^**	**50 *μ*M Novobiocin^a^**	**100 *μ*M Novobiocin^a^**	**Efflux^b^**
482G-1	65.3±7.6	107.8±9.9	221.9±9.8	161.1±8.6	264.3±18.3
482G-2	90.5±15.7	116.2±15.7	279.6±35.1	199.9±19.0	301.4±9.1
482R-2	44.9±2.5	73.6±1.4	97.3±13.6	47.6±2.0	163.0±10.3
482R-5	62.8±3.9	82.2±2.5	132.6±24.6	58.0±8.9	218.2±11.1
482T-7	78.5±48.8	88.2±9.9	279.3±13.7	242.4±57.5	284.0±26.3
482T-10	87.4±5.7	104.8±20.2	236.8±9.0	216.6±38.5	263.1±8.7

a The values shown are the difference in mean channel numbers between the FTC/efflux histogram and the inhibitor/efflux histogram of the noted inhibitor.

b Difference in mean channel number between the FTC/efflux histogram and the efflux histogram. Values were obtained from two separate experiments.

were generated by subtracting the mean channel number of the inhibitor/efflux histogram from the FTC/efflux histogram. The more effective the inhibitor, the smaller the difference between the inhibitor/efflux histogram and the FTC/efflux histogram. Values in the efflux column were obtained by subtracting the mean channel value of the efflux histogram from the FTC/efflux histogram, providing a measure of ABCG2 activity in each cell line. Both of the 482R clones show slightly lower BODIPY-prazosin efflux than the mutant clones, a result that parallels the mitoxantrone cross-resistance and transport data. The compounds *β*-estradiol and estrone (Imai *et al*, 2002) have also recently been reported to inhibit ABCG2-mediated transport. We confirm that both compounds do block ABCG2, although *β*-estradiol appears to be more effective than estrone.

**Figure 4:**
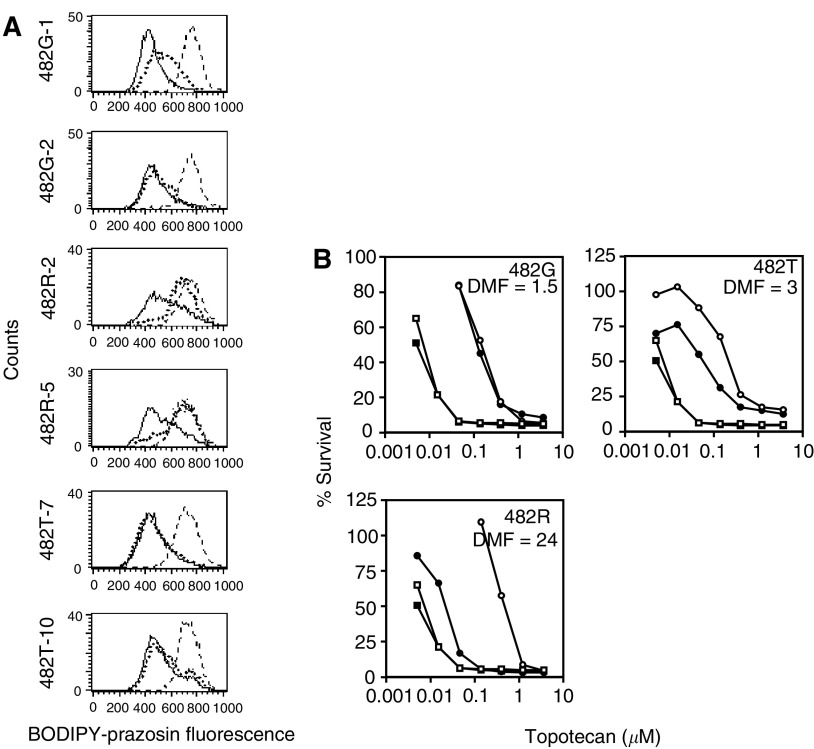
Novobiocin reverses wild-type ABCG2 but not mutant ABCG2-mediated resistance. (**A**) The six transfected cell lines were incubated in 250 nM BODIPY-prazosin with or without 50 *μ*M novobiocin for 30 min at 37°C, washed, then allowed to efflux for 1 h at 37°C continuing with (dotted line) or without (solid line) novobiocin and compared to cells incubated in 10 *μ*M FTC (dashed line). (**B**) The 4-day cytotoxicity assays were performed with topotecan with (filled symbols) or without (open symbols) 50 *μ*M novobiocin on HEK-293 cells expressing wild-type (482R) or mutant (482G, 482T) ABCG2 as detailed in the Materials and Methods section. Representative results are shown. Empty vector transfected cells are shown as squares while ABCG2 transfected cells are denoted by circles. Dose-modifying factor values were obtained for the ABCG2-transfected cells by dividing the IC_50_ for topotecan without novobiocin by the IC_50_ value for topotecan in the presence of novobiocin.

The ability of the putative ABCG2 antagonist novobiocin (Doyle *et al*, 2002; Shiozawa *et al*, 2002) to block ABCG2-mediated efflux of BODIPY-prazosin was most affected by the amino acid at position 482. The difference between the 50 *μ*M novobiocin/efflux histogram and the FTC/efflux histogram was smallest for cells transfected with wild-type ABCG2, suggesting that novobiocin is more effective in inhibiting wild-type-mediated transport, as shown in Table 3. Efflux mediated by the mutant ABCG2 proteins was nearly unaffected by novobiocin. Increasing the concentration of novobiocin to 100 *μ*M almost completely abrogated wild-type ABCG2-mediated efflux. Histograms for the transfectants are shown in Figure 4A

. BODIPY-prazosin fluorescence in the presence of 50 *μ*M novobiocin (dotted line) in HEK-293 cells transfected with wild-type ABCG2 was comparable to that in cells incubated with 10 *μ*M FTC (dashed line). For 482R-5, these histograms overlap. However, novobiocin was less effective in mutant 482G and 482T ABCG2, where BODIPY-prazosin accumulation with novobiocin is near that of cells incubated with BODIPY-prazosin alone (solid line).

To verify the flow cytometry results, 4-day cytotoxicity assays were then performed on HEK-293 cells transfected with wild-type and mutant ABCG2 in the presence of topotecan with (filled symbols) and without (open symbols) 50 *μ*M novobiocin as shown in Figure 4B. Topotecan was selected because cross-resistance studies (in Table 2) suggested that cells transfected with wild-type ABCG2 exhibited slightly higher resistance to the drug compared to the mutant proteins. In empty vector transfected cells (squares) as well as the mutant 482G-1 and 482T-10 cells (circles), 50 *μ*M novobiocin had little effect on topotecan cytotoxicity. Cells transfected with 482G ABCG2 were 21-fold resistant to topotecan (filled circles) and 14-fold resistant in the presence of 50 *μ*M novobiocin (open circles), while cells transfected with 482T ABCG2 were 29-fold resistant to topotecan (filled circles) and were nine-fold resistant to the drug in the presence of novobiocin (open circles). In contrast, HEK-293 cells transfected with wild-type ABCG2 (482R) were 71-fold resistant to topotecan (filled circles), and were only three-fold resistant to the drug in the presence of 50 *μ*M novobiocin. Thus, novobiocin nearly completely reversed wild-type ABCG2-mediated resistance to topo-tecan. Dose-modifying factors (DMFs) were also calculated for each transfectant by dividing the IC_50_ for topotecan alone by the IC_50_ of topotecan in the presence of novobiocin and are given in Figure 4B.

## DISCUSSION

To confirm earlier transport and resistance studies conducted with selected cell lines overexpressing wild-type and mutant ABCG2 (Honjo *et al*, 2001; Robey *et al*, 2001a), HEK-293 cells were transfected with vectors encoding wild-type or mutant ABCG2. Our results with these transfectants confirm that the fluorescent compounds mitoxantrone, BODIPY-prazosin, and Hoechst 33342 are substrates of all three ABCG2 proteins tested in the present study, while LysoTracker Green, daunorubicin, and rhodamine 123 are only appreciably transported by the mutant 482G and 482T proteins. In cytotoxicity assays, all ABCG2 proteins conferred high levels of resistance to mitoxantrone, topotecan, and SN-38, while the mutant proteins conferred four times more resistance to mitoxantrone than the wild-type protein. Cells transfected with wild-type ABCG2 appeared slightly more resistant to SN-38 and topotecan. The mutant 482G and 482T ABCG2 proteins conferred 10- to 71-fold resistance to the Pgp substrates doxorubicin, daunorubicin, and epirubicin. In contrast, wild-type 482R ABCG2 conferred only three- to four-fold resistance to these agents. Thus, while flow cytometric analysis suggests no transport of daunorubicin or rhodamine 123 in cells transfected with wild-type ABCG2, cross-resistance studies do suggest that wild-type ABCG2 confers a very low level of resistance to these two compounds. All three ABCG2 proteins conferred low levels of resistance to etoposide, while no appreciable resistance to paclitaxel, depsipeptide or flavopiridol was noted.

Mutations at amino-acid 482 have included R482G and R482T in human cancer cells; R482S and R482M in mouse fibroblast lines (Honjo *et al*, 2001; Allen *et al*, 2002); and a recently reported R482M mutation in a doxorubicin-selected human T-cell line (Wang *et al*, 2003). Since amino-acid 482 is predicted to be at the beginning of the third transmembrane segment on the intracellular surface of the membrane, it could be directly involved in substrate binding or could affect substrate transport through structural alterations in the third transmembrane segment (Honjo *et al*, 2001). Although Allen *et al* (2002) have suggested that loss of the basic arginine at amino-acid 482 may be critical to substrate specificity, mechanistic studies are needed to understand the changes that result from alteration at amino-acid 482.

Since the wild-type arginine is a bulky, positively charged amino acid, it could impede binding or transport of positively charged compounds. While this could explain the poor transport of doxorubicin and daunorubicin, both of which possess aliphatic amino groups that are positively charged at a physiological pH (7.6), we would note that mitoxantrone, a substrate for all ABCG2 proteins, has a similar structure and charge. LysoTracker and BODIPY-prazosin both carry a positive charge on a boron atom, thus resembling rhodamine's positively charged amino group, yet only prazosin is a substrate for all three ABCG2 proteins. These observations suggest that a positive charge alone is not predictive of whether a compound will be an ABCG2 substrate.

Resistance to the anionic methotrexate has also recently been shown to be affected by mutations in ABCG2 at amino-acid 482. Volk *et al* report that selected cell lines expressing wild-type ABCG2 are resistant to methotrexate. Loss of the positive charge at amino-acid 482 by mutation to glycine or threonine yields a protein conferring much less resistance to the drug (Volk *et al*, 2002). It seems reasonable to assume that mutations at amino-acid 482 in ABCG2 could affect the ability of the protein to confer resistance to other drugs currently being evaluated for use in the clinic.

The findings presented here parallel those of Allen *et al*, (2002), who described two mutations, R482M and R482S, in mouse fibroblast cells lacking functional Mdr1, Mdr2, and Mrp1. These authors observed greater anthracycline resistance, lower topotecan resistance and enhanced transport of rhodamine 123. Unlike our observations in human cells where a change from arginine to glycine or threonine occurred early in the course of the drug selection, in the mouse fibroblasts selected with doxorubicin, overexpression and amplification of wild-type *Abcg2* occurred before mutation at amino-acid 482. This would suggest that murine Abcg2 can confer biologically meaningful resistance to doxorubicin; a finding that appears to be in conflict with the very low levels of resistance we observed in HEK-293 cells transfected with wild-type human ABCG2. This may simply be due to differing substrate affinities in the ABCG2 protein in the two species, as has been reported for MRP1 (Stride *et al*, 1997).

Mutations at amino-acid 482 also were found to alter the efficacy of a reported ABCG2 inhibitor. Novobiocin, a compound recently reported to block ABCG2-mediated efflux (Doyle *et al*, 2002; Shiozawa *et al*, 2002), was found to be most effective on wild-type ABCG2 and nearly ineffective on mutant ABCG2. This would suggest that, if the described R482T or R482G mutations in ABCG2 were to occur in patients, they could render currently known ABCG2 inhibitors less effective. FTC at 10 *μ*M appeared to inhibit all of the ABCG2 proteins equally well. Both *β*-estradiol and estrone inhibited ABCG2-mediated prazosin transport; however, estrone was the least effective of the two.

It was surprising that the transfected cells displayed no resistance to flavopiridol, since we have reported overexpression of ABCG2 in cells selected with flavopiridol and have shown cross-resistance to flavopiridol in ABCG2 overexpressing selected cell lines (Robey *et al*, 2001b). This could be due to the fact that flavopiridol is a substrate with a weak affinity to ABCG2 and thus the expression of ABCG2 in the transfected cells is inadequate to confer appreciable resistance. In support of this explanation is the cross-resistance data obtained in a cell line selected in flavopiridol (MCF-7 FLV1000) which was only 24-fold resistant to flavopiridol but was 675-fold resistant to mitoxantrone. Alternatively, modification of flavopiridol may be necessary to allow it to be better transported. Recent studies have shown that flavopiridol is most likely glucuronidated in human liver by UDP-glucuronosyltransferase isoforms (Ramirez *et al*, 2002). We have previously suggested a link between glucuronidation and resistance mediated by ABCG2, as we noted increased expression of UDP-glucuronosyltransferase in cell lines that overexpress ABCG2 (Brangi *et al*, 1999). It is thus possible that breast carcinoma cells selected in flavopiridol have the capacity to glucuronidate flavopiridol, while HEK-293 cells do not. Studies are currently underway to determine if flavopiridol-selected cells do indeed glucuronidate flavopiridol in order to transport it.

In summary, we show that mutation of a single amino acid in the ABCG2 protein has a major effect on its substrate specificity, and may possibly undermine the effectiveness of potential ABCG2 blockers.
